# Occludin regulates HIV-1 infection by modulation of the interferon stimulated OAS gene family

**DOI:** 10.21203/rs.3.rs-2501091/v1

**Published:** 2023-01-30

**Authors:** Silvia Torices, Timea Teglas, Oandy Naranjo, Nikolai Fattakhov, Kristyna Frydlova, Rosalba Cabrera, Olivia M. Osborne, Enze Sun, Allan Kluttz, Michal Toborek

**Affiliations:** University of Miami Miller School of Medicine: University of Miami School of Medicine; University of Miami Miller School of Medicine: University of Miami School of Medicine; University of Miami Miller School of Medicine: University of Miami School of Medicine; University of Miami Miller School of Medicine: University of Miami School of Medicine; University of Miami Miller School of Medicine: University of Miami School of Medicine; University of Miami Miller School of Medicine: University of Miami School of Medicine; University of Miami Miller School of Medicine: University of Miami School of Medicine; University of Miami Miller School of Medicine: University of Miami School of Medicine; University of Miami Miller School of Medicine: University of Miami School of Medicine; University of Miami School of Medicine

**Keywords:** OAS, occludin, RNaseL, interferon, HIV-1, pericytes, blood brain barrier

## Abstract

HIV-1-associated blood brain barrier (BBB) alterations and neurocognitive disorders are frequent clinical manifestations in HIV-1 infected patients. The BBB is formed by cells of the neurovascular unit (NVU) and sealed together by tight junction (TJ) proteins, such as occludin (ocln). Pericytes are a key cell type of NVU that can harbor HIV-1 infection via a mechanism that is regulated, at least in part, by ocln. After viral infection, the immune system starts the production of interferons, which induce the expression of the 2’−5’-oligoadenylate synthetase (OAS) family of interferon stimulated genes and activate the endoribonuclease RNaseL that provides antiviral protection by viral RNA degradation. The current study evaluated the involvement of the OAS genes in HIV-1 infection of cells of NVU and the role of ocln in controlling OAS antiviral signaling pathway. We identified that ocln modulates the expression levels of the OAS1, OAS2, OAS3, and OASL genes and proteins and, in turn, that the members of the OAS family can influence HIV replication in human brain pericytes. Mechanistically, this effect was regulated via the STAT signaling. HIV-1 infection of pericytes significantly upregulated expression of all OAS genes at the mRNA level but selectively OAS1, OAS2 and OAS3 at the protein level. Interestingly no changes were found in RNaseL after HIV-1 infection. Overall, these results contribute to a better understanding of the molecular mechanisms implicated in the regulation of HIV-1 infection in human brain pericytes and suggest a novel role for ocln in controlling of this process.

## Introduction

Infection by Human Immunodeficiency Virus (HIV) affects millions of people around the world [1]. HIV alters the integrity of the blood brain barrier (BBB) early in the course of infection and enters the brain, where the infection remains persistent [2–6]. The BBB is a thoroughly selective physiological interphase that allows for the division of systemic blood circulation from the brain parenchyma. While endothelial cells (EC) are the primary cell type forming the microvessels, astrocytes, neurons, pericytes, and microglia cells coordinate their functions with EC by forming the neurovascular units (NVU) [7], the structural elements of the BBB.

Among the cells of the NVU, pericytes have recently attained importance for their ability to regulate the integrity, maintenance, and development of the BBB [8–11]. Importantly, pericytes express CD4 and chemokine coreceptors, which allow them to be directly infected by HIV [12, 13]. Indeed, several studies confirmed that BBB pericytes can harbor active HIV-1 infection, and potentially can function as reservoirs of the virus [6, 12–17]. It has been also proposed that latent HIV-1 infection in pericytes can be reactivated and release the virus into the CNS [6, 14–16]. Pericytes can propagate cellular dysfunction after HIV-1 infection via gap junction-mediated intercellular communication [18]. During HIV-1 infection, various functional and structural alterations of the BBB have been associated with altered expression of tight junction (TJ) proteins, including occludin (ocln) [14, 16, 19, 20]

Ocln is a tetraspan redox-sensitive protein associated with tight junctions of the BBB, and plays a key role in maintaining the integrity of the BBB [21]. Ocln is ubiquitously throughout different cell types including pericytes. Research from our laboratory has indicated that ocln influences cellular metabolism through AMPK protein kinase activity [22]. Moreover, ocln has been shown to function as a NADH oxidase, whereby increasing the expression of NAD + and regulating SIRT-1 levels [14]. Prominently, data from human pericytes and other cell types, such as macrophages support the notion that ocln can regulate the extent of HIV-1 infection [14, 16]. Given its capacity to indirectly regulate acetylation and phosphorylation, ocln levels were correlated with amended expression of several proteins responsible for maintaining the integrity of the BBB and HIV-1 infection [16]. However, the mechanisms involved in the regulatory impact of ocln on HIV-1 infection remain unclear.

After viral infection, the immune system starts the production of antiviral cytokines, with interferons (IFNs) [15] being the most prominent. The 2’, 5’- oligoadenylate synthetases (OAS) are the family of INF-stimulated genes that play a significant role in innate immune response. OAS proteins have been described to have antiviral functions by acting as nucleotidyltransferases, catalyzing the oligomerization of ATP into 2’, 5- linked oligoadenylates (2–5A), which leads to the activation of latent RNaseL. RNaseL provides antiviral protection via degradation of viral RNA [23–28]. Four genes, OAS1, OAS2, OAS3 and OAS-like (OASL) located on chromosome 12 have been identified as the members of the human OAS gene family, and 10 isoforms, including OAS1 (p42, p44, p46, p48, and p52), OAS2 (p69, and p71), OAS3 (p100), and OASL (p30 and p59) are generated by alternative splicing of these genes [29–34]. Human OAS1 forms a tetramer, OAS2 a dimer, and OAS3 forms a monomer. OASL has been reported to lack OAS activity, but instead it can activate antiviral retinoic acid-inducible gene 1 (RIG-1) signaling after dsRNA infection [31, 32, 35–38]. The location, induction, and enzymatic parameters of the OAS proteins can vary between different cell types [29]. In the mouse genome, eight OAS1, one OAS2, and OAS3 have been described on chromosome 8, and two OASL on chromosome 5 [39, 40]. The expression of the OAS family is unknown in human pericytes; therefore, the goal of the present study was to evaluate the profile of the members of the OAS family in cells of the NVU and assess their antiviral function in the context of HIV-1 infection of brain pericytes.

Overall, we describe for the first time the expression of the OAS proteins in cells forming the NVU, with the focus on their role in brain pericytes. The expression of the OAS family members is dependent on ocln expression via altering the STAT signaling pathway response. Moreover, the OAS genes effectively influence HIV-1 replication in pericytes. Collectively, our findings indicate that ocln is a critical component in controlling immune responses due to its ability to regulate the expression of the OAS genes and proteins.

## Material And Methods

### Cell Cultures

Human embryonic kidney (HEK)-293T cells (ATCC, Manassas, VA, USA, Cat# CRL-11268) were cultured in DMEM (Thermo Fisher Scientific, Carlsbad, CA, USA, Cat#11995–065) supplemented with 10% of FBS (ScienCell, Cat# 0500), 100 μg/mL of streptomycin and penicillin 100 units/mL (Thermo Fisher Scientific, Cat# 15140–122). Primary human brain vascular pericytes (ScienCell, Carlsbad, CA, USA, Cat# 1200) from six different lots (passages 2–7) were cultured in pericyte-specific growth medium (ScienCell, Cat# 1201) supplemented with 2% of fetal bovine serum (FBS), growth factors, 100 μg/mL of streptomycin and 100 units/mL of penicillin. Primary human astrocytes (ScienCell, Carlsbad, CA, USA, Cat #1800) were cultured in an astrocyte-specific growth medium (ScienCell, #1801) supplemented with 2% FBS, astrocyte growth supplement, 100 units/mL penicillin, and 100 μg/mL streptomycin. Primary human brain microvascular endothelial cells were obtained from Cell Systems (Kirkland, WA, USA, Cat #ACBRI 376) and cultured in a medium supplemented with CultureBoost, 10% serum, 100 units/mL penicillin, and 100 μg/mL streptomycin. Immortalized human microglia cell line (hpglia C20) was kindly provided by Dr. Jonathan Karn (Case Western Reserve University, Ohio, OH, USA). Hpglia C20 cells were generated by SV40/hTERT-mediated immortalization [41] and cultured as described [42] in BrainPhys medium (StemCell Technologies, Vancouver, BC, Canada, Cat# 05791) containing 1X N2 supplement-A (Thermo Fisher Scientific, Cat #17502–048), 1X penicillin streptomycin (Gibco, Cat #15140122), 100 μg/mL normocin (InvivoGen, San Diego, CA, USA, Cat #ant-nr-1), 25 mM L-Glutamine (Thermo Fisher Scientific, Cat#25030081), 1% FBS, and 1 μM dexamethasone (Sigma-Aldrich, St. Louis, MO, USA Cat #D4902). Human neuroblastoma SH-SY5Y cell line (ATCC, Cat#CRL-2266) was cultured in Dulbecco’s Modified Eagle Medium (DMEM) (Thermo Fisher Scientific, Carlsbad, CA, USA, Cat#11995–065) and supplemented with 10% FBS (ScienCell, Cat# 0500), 100 units/mL penicillin, and 100 μg/mL streptomycin (Thermo Fisher Scientific, Cat# 15140–122). All cultures were maintained at 37°C in 5% of CO_2_.

### HIV-1 stock preparation

HIV pNL4–3 plasmid obtained from the NIH AIDS Reagent Program (Division of AIDS, NIAID, National Institutes of Health) was employed for human infection. HIV pNL4–3 plasmids was amplified using Stbl3 competent cells (Thermo Fisher Scientific, Cat# C737303) and isolated using PureYield Plasmid midi-prep system (Promega, Madison, WI, USA, Cat# A2492). Viral stocks were created as described [16]. Briefly, a total of 50 μg of proviral plasmid was transfected into 10^7^ human HEK293T/17 cells using Lipofectamine 2000 (Thermo Fisher Scientific, Cat# 11668–027). Media was changed to fresh Opti-Mem (Thermo Fisher Scientific, Cat# 11058–021) 18 hours post transfection and cells were incubated for additional 48 hours. Supernatant was then collected and filtered through 0.45 μm-pore size (Millipore Sigma, Massachusetts, MA, USA, Cat# 430314) filters to remove cell debris. Supernatants were concentrated using 50 kDa molecular weight exclusion columns (Millipore Sigma, Cat# UFC905024) and the aliquots were stored at −80°C.

### HIV-1 infection

For *in vitro* HIV-1 infection, pericytes were incubated with a total of 60 ng/ml of HIV-1 p24, which was followed by extensive washing with PBS to remove the unbound virus before addition of fresh medium. HIV infection rates were quantified by the assessment of p24 levels using HIV-1 p24 Antigen ELISA 2.0 assay (Zeptometrix, Buffalo, NY, USA, Cat# 0801008) following the manufacturer’s instructions. The levels of p24 were calculated in pg/ml.

### Gene overexpression and silencing

Pericytes were transfected using Amaxa Nucleofector Technology and the Basic Nucleofector Kit (Lonza, Switzerland, EU, Cat# VPI-1001) as described [16]. For ocln overexpression, cells were transfected with 1 μg PCMV3-OCLN plasmid (Sino Biological, Wayne, PA, Cat# HG15134-UT) or with PCMV3 (Sino Biological, Cat# CV011) as a negative control. For gene silencing, cells were transfected with 0.5 μg per 10^6^ cells of the following small interfering RNA (siRNA): 3 unique 27mer OAS1-siRNAs (OriGene, Rockville, MD, USA, Cat# SR303266), 3 unique 27mer OAS2-siRNAs (OriGene, Cat# SR303267), 3 unique 27mer OAS3-siRNAs (OriGene, Cat# SR303268), 3 unique 27mer OASL-siRNAs (OriGene, Cat# SR305676), 3 unique 27mer RNaseL-siRNAs (OriGene, Cat# SR304081), 3 unique 27mer occludin-siRNAs (OriGene, Cat# SR303274), 3 unique 27mer TJP1-siRNAs (OriGene, Cat# SR322042), or trilencer-27 universal scrambled (SCR) silencer siRNA duplex (OriGene, Cat# SR30004) as a negative control.

### Quantitative real-time PCR (qPCR)

qPCR was performed using Applied Biosystems 7500 system (Applied Biosystems, Foster City, CA). Briefly, mRNA isolation from human brain pericytes was performed using the RNeasy mini-kit (Qiagen, Cat# 74104) according to the manufacturer’s instructions. Total RNA was quantified using Nanodrop 2000 (Thermo Fisher Scientific). A total of 100ng of RNA was used in each reaction. Reverse transcription and qPCR reactions were performed using the qScript XLT 1-Step RT-qPCR Tough Mix (Quantabio, Beverly, MA, USA, Cat #89236–676). The primers used for gene amplification by TaqMan Gene Expression Assays are listed in supplementary Table 1. Specificity of qPCR results was established using melting curve assessment, and gene expression fluctuations were determined by the ΔΔCt method, with Ct as the cycle number at threshold. The results were normalized to GAPDH expression.

### Protein extraction and immunoblotting [43]

For WB, human pericytes were lysed with 100 μl/well of RIPA buffer including protease inhibitors (Santa Cruz Biotechnology, Dallas, TX, USA, Cat# sc-24948a). Protein concentrations were measured by BCA Protein Assay Kit (Thermo Fisher Scientific, Cat# 23223) according to the manufacturer’s instructions. Proteins were separated using 4–20% Mini-PROTEAN TGX Stain-Free Protein Gels and the PVDF membrane Trans-Blot Turbo Transfer System (Bio-Rad Laboratories, Hercules, CA, USA, Cat# 170–4159) and blocked with 5% bovine serum album (BSA) in TBS-0.05% Tween20 for 1 hour. Subsequently, blots were incubated overnight at 4°C with the following primary antibodies: anti-occludin (Thermo Fisher Scientific, Cat# 71–1500), anti-OAS1 p46 (Proteintech, Rosemont, IL, USA, Cat# 14955–1-AP), anti-OAS2 p69/71 (Thermo Fisher Scientific, Cat# MA5–26552), anti-OAS3 p100 (Thermo Fisher Scientific, Cat# PA5–59539), anti-OASL p59 (Thermo Fisher Scientific, Cat# PA5–81946), anti-RNaSeL (Cell Signaling, Danvers, MA, USA, Cat# 27281), anti-STAT1 (Cell Signaling Technology, Cat# 9172), anti-phospho-STAT1 (Tyr701) (Cell Signaling Technology, Cat# 9171), or anti-STAT2 (Cell Signaling Technology, Cat# 4594). All antibodies were used at 1:1000 in 5% BSA-TBS. After washing with TBS-0.05% Tween20 three times for 5 min, samples were incubated for 1 hour with the secondary antibodies (LI-COR, Lincoln, NE, USA, Cat# 926–32210, Cat# 926–68070, and Cat# 926–32211, Cat # 926–68071) at 1:20000 in 5% BSA-TBS and analyzed using the Licor CLX imaging system and the Image Studio 4.0 software (LI-COR). Anti-GAPDH antibody (1:20000, Novus Biologicals, Woburn, MA, USA, Cat# NB600–502FR or Cat# NB600–5021R) was used for protein normalization.

### Luciferase assay

Human brain pericytes were co-transfected with 1 μg of PCMV3-OCLN plasmid, 2 μg of firefly luciferase construct under the control of the STAT1 promoter Pgl4.45 (luc2P/ISRE/Hygro plasmid; Promega, Madison, WI, USA, Cat# E414A), and 0.5 μg of the pRL Renilla Luciferase Control Reporter Vector (Promega, Cat# E2231). Cell lysates were analyzed using the dual-luciferase reporter assay (Promega, Cat# E1910). Renilla luciferase fluorescence levels were used to normalize the luciferase fluorescence.

### Statistical Analysis

Statistical analyses were achieved utilizing GraphPad Prism Software (La Jolla, CA, USA). The significance level was placed at *p* < 0.05 (****p < 0.0001, ***p = 0.0002, **p = 0.003, *p < 0.0449). Statistical significance was established by one-way or two-way ANOVA followed by Turkey’s multiple comparisons test or Student’s t test.

## Results

### OAS are differentially expressed in the cells of the NVU

While an association was demonstrated between the OAS gene expression and the progression of several viral infections and autoimmune diseases [44–47], no studies have characterized the expression of the members of the OAS family in human brain or brain microvasculature. Here, we describe the protein expression levels of OAS1, OAS2, OAS3, and OASL in cells forming the NVU, such as primary human brain pericytes, astrocytes, EC, immortalized human microglial cells, and SH-SY5Y neuroblastoma cell line.

**Figure 1:**
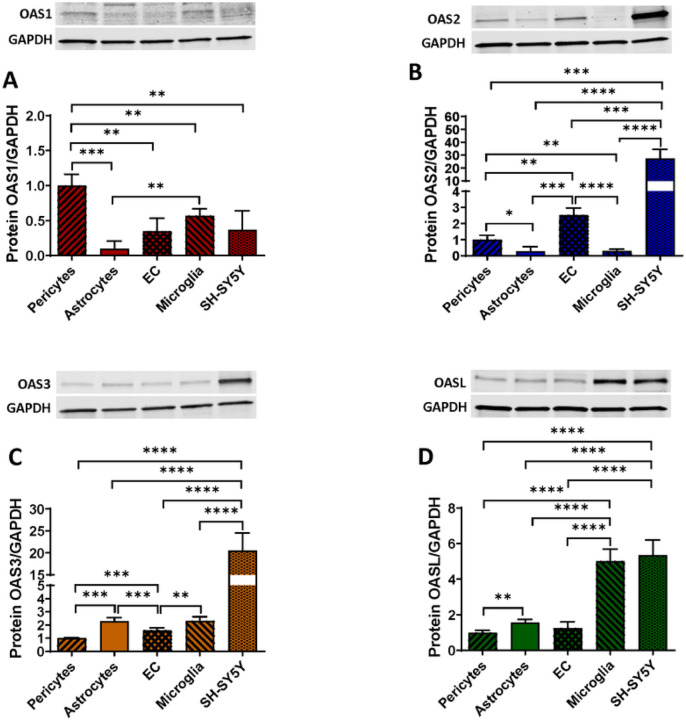
Expression pattern of the OAS proteins in cells of the NVU. Expression of OAS1 **(A)**, OAS2 **(B)** OAS3 **(C)** and OASL **(D)** in primary human brain pericytes, astrocytes, EC, immortalized human microglial cells, and SH-SY5Y neuroblastoma cell line as measured by immunoblotting. GAPDH was used as a loading control. Values are mean ± SEM. ****p < 0.0001, ***p = 0.0002, **p = 0.003, *p < 0.0449, n = 4 independent samples per group.

Among the cells of NVU, pericytes expressed the highest levels of OAS1, followed by microglia, EC, and SH-SY5Y. The lowest expression of OAS1 was found in astrocytes (Fig. 1A) In the case of OAS2, SH-SY5Y cells expressed significantly higher levels than other studied cells, followed by EC, and pericytes. Microglia and astrocytes expressed the lowest levels of OAS2 (Fig. 1B) For OAS3, the highest expression was found in SH-SY5Y cells, followed by significantly lower expression in astrocytes and microglia (Fig. 1C). The lowest levels of OAS3 were found in EC and pericytes. Lastly, the highest levels of OASL proteins were detected in microglia and SH-SY5Y cells, with significant lower expression in astrocytes, EC, and pericytes (Fig. 1D). Overall, these results indicate a differential expression pattern of the OAS family members in cells composing the NVU, suggesting that these cells are involved in antiviral protection.

### Ocln regulates IFN genes and alters the STAT signaling pathway

**Figure 2:**
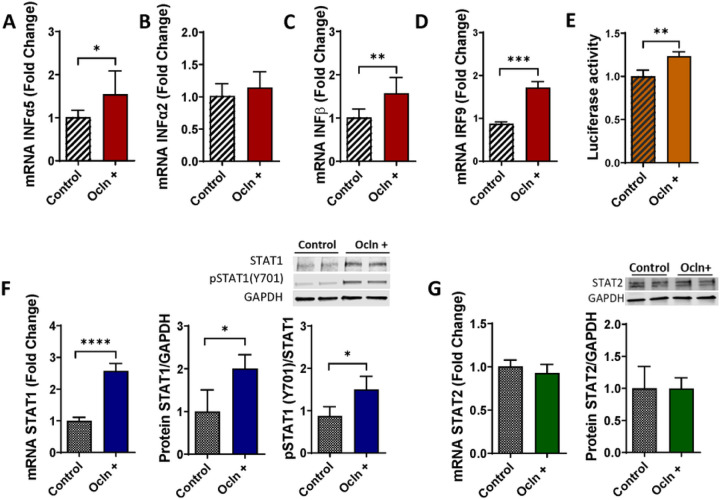
Ocln regulates IFN genes and alters the STAT signaling pathway. Pericyte ocln levels were overexpressed by transfection with PCMV3-OCLN expression vector, followed by the assessment of the mRNA of INFα5 **(A)**, INFα2 **(B)** and INFβ **(C)**. INFγ expression was non-detectable. mRNA and protein of STAT1, STAT1 Tyr 701 phosphorylation **(F)** and STAT2 **(G)** were measured. **(E)**Cells were co-transfected with PCMV3-OCLN plasmid and a luciferase construct under the control of the STAT1 promoter and cell lysates were analyzed using the dual-luciferase reporter assay. In addition, IRF9 **(D)** were assessed by q-PCR. Graphs indicate the mean ± SEM from three independent experiments. ****p < 0.0001, ***p = 0.0002, **p = 0.003, n = 4–9 per group.

Ocln has been traditionally consider as a tissue barrier regulating protein; however, recent evidence indicated multifunctional role of this protein in controlling cellular metabolism and HIV-1 infection [14, 16, 22]. Therefore, we evaluated the impact of ocln on mRNA and protein expression of the IFN genes as the main component of innate immunity. These experiments focused on pericytes as the NVU cells, which can harbor HIV-1 infection [6, 13, 16, 18]. Pericytes were transfected with the PCMV3-OCLN expression vector, and the expression levels of several IFN genes were evaluated by real time q-PCR. The results indicated that ocln overexpression led to significantly increased IFNa5 (Fig. 2A), and IFNp (Fig. 2C). In contrast, no significant changes were found in IFNa2 (Fig. 2B) and the expression of IFNy where not detectable.

We next analyzed the STAT signaling pathway that initiate the transcription of IFN-stimulated genes (ISGs) [48, 49]. One of the key elements in the STAT signaling pathway are the signal transducer and activator of transcription (STAT)1 and STAT2 proteins, which upon phosphorylation, translocate into the nucleus where they initiate the transcription of ISGs. Therefore, we examined if modulations of ocln levels can alter the expression of STAT proteins. The results indicate that ocln overexpression markedly induced the expression of STAT1 at the gene and protein levels but not STAT2 (Fig. 2F–G). Moreover, ocln overexpression led to an increase in phosphorylated STAT1 (pSTAT1) at Tyr701 (Fig. 2F). To analyze the functional consequences of ocln upregulation on the activity of STAT1, cells were co-transfected with firefly luciferase constructs under the control of the STAT1 promoter. As shown in Fig. 2E, ocln upregulation led to an increase in STAT1 binding activity. Moreover, interferon regulatory factors (IRFs) are molecules that execute positive feedback with type I IFN. For example, activated STAT1 recruits IRF9 forming a complex that translocate to the nucleus. Consistent with this mechanism, ocln overexpression resulted in upregulation of IRF9 gene expression (Fig. 2D).

### Ocln regulates OAS expression levels

**Figure 3:**
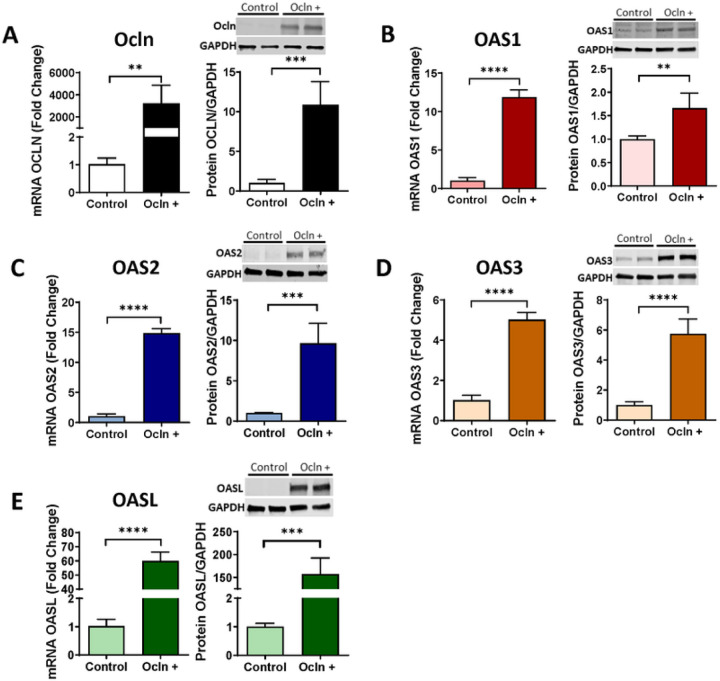
Ocln upregulation enhances OAS expression in human brain pericytes. Pericytes were transfected with ocln overexpression vector PCMV3-OCLN or with PCMV3 control vector, and the expression of mRNA and protein for ocln (A) OAS1 (B) OAS2 (C) OAS3 (D) and OASL (E) was evaluated by q-PCR and immunoblotting respectively. GAPDH was used as a housekeeping gene and loading control. Values are mean ± SEM. ****p < 0.0001, ***p = 0.0002, **p = 0.003, *p < 0.05, n = 4–6 independent samples per group.

We next focused on the OAS genes and protein family as a prominent component of native immunity regulated by IFN. As in Figure 2, pericytes were transfected with the PCMV3-OCLN vector for ocln overexpression or with the PCMV3 vector as a negative control, and the expression of ocln, OAS1, OAS2, OAS3 and OASL was analyzed by qPCR and immunoblotting. Ocln overexpression led to remarkably significant increase in OAS1, OAS2, OAS3, and OASL mRNA and protein (Fig. 3A–E, respectively) levels. Interestingly, this increase was notably higher for OASL when compared to OAS1, OAS2 or OAS3.

**Figure 4:**
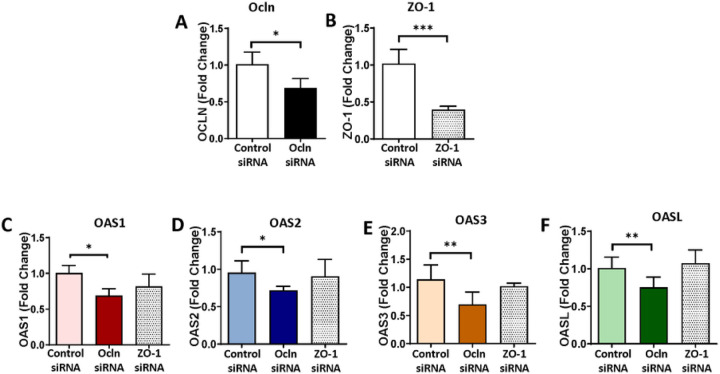
OAS expression is specifically regulated by ocln and not altered by silencing of tight junction protein ZO-1. Pericytes were transfected with ocln-siRNA, ZO-1 siRNA, or control-siRNA, and the expression of genes encoding for ocln **(A)** ZO-1 **(B)** OAS1 **(C)** OAS2 **(D)** OAS3 **(E)** and OASL **(F)** was evaluated by q-PCR. GAPDH was used as a housekeeping gene. Values are mean ± SEM. ****p < 0.0001, ***p = 0.0002, **p = 0.003, *p < 0.05, n = 4–6 independent samples per group.

In the next series of experiments, we measured the expression of the OAS family in pericytes with silenced ocln gene. The controlled experiments were performed with silenced ZO-1, another tight junction protein to determine specificity of ocln-mediated responses. Briefly, pericytes were transfected with control siRNA, ocln siRNA, or ZO-1 siRNA, and the expression of the OAS genes was analyzed by q-PCR. Ocln silencing (Fig. 4A) but not ZO-1 silencing (Fig. 4B) resulted in a significant decrease in the expression of OAS1, OAS2, OAS3, and OASL mRNA (Fig. 4C–F). Along with Figure 3, these results indicate a regulatory influence of ocln on the expression of the members of the OAS family. They also suggest a novel mechanism by which ocln can influence innate immunity and protect against viral infection.

### OASL, but no other members of the OAS family, alters ocln expression levels

**Figure 5:**
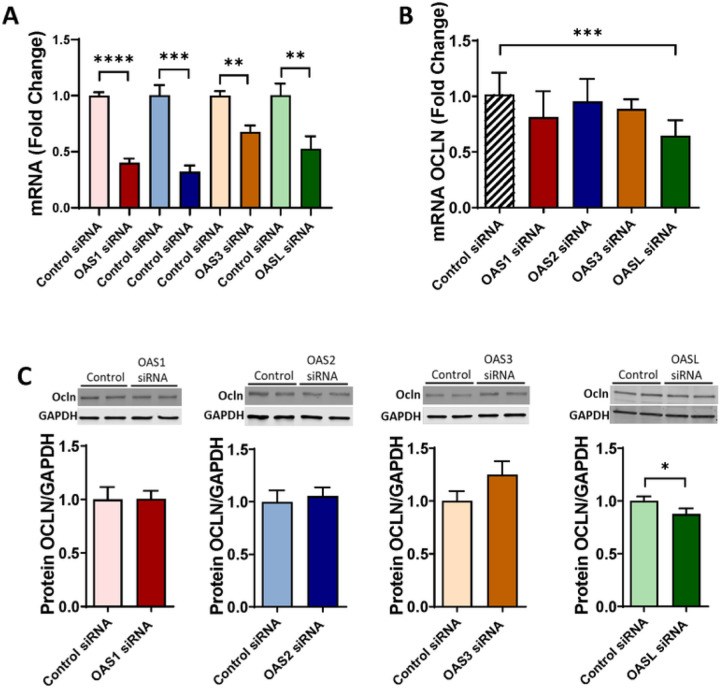
Ocln expression is specifically regulated by OASL and not by other members of the OAS family. Pericytes were transfected with OAS1 siRNA, OAS2 siRNA, OAS3 siRNA, OASL siRNA, or control-siRNA **(A)**, and ocln expression was evaluated by q-PCR **(B)**and immunoblotting **(C)**. GAPDH was used as a housekeeping gene and loading control. Values are mean ± SEM. ****p < 0.0001, ***p = 0.0002, **p = 0.003, *p < 0.05, n = 4–8 independent samples per group.

Because ocln can modify the expression of the OAS genes, we next investigated if the reverse modulation can also occur. Pericytes were transfected with control siRNA, OAS1 siRNA, OAS2 siRNA, OAS3 siRNA, or OASL siRNA, and ocln mRNA and protein expression levels were measured by qPCR and immunoblotting, respectively. The efficiency of silencing of the individual OAS genes was confirmed by qPCR (Fig. 5A). We then measured ocln expression in these samples. Among studied OAS genes, only OASL silencing led to a relatively small but a significant decrease in ocln levels at mRNA (Fig. 5B) and protein levels (Fig. 5C) contrast, no changes in ocln expression were found after silencing the remaining members of the OAS family.

### Cross-regulation of the expression among the OAS family members

**Figure 6:**
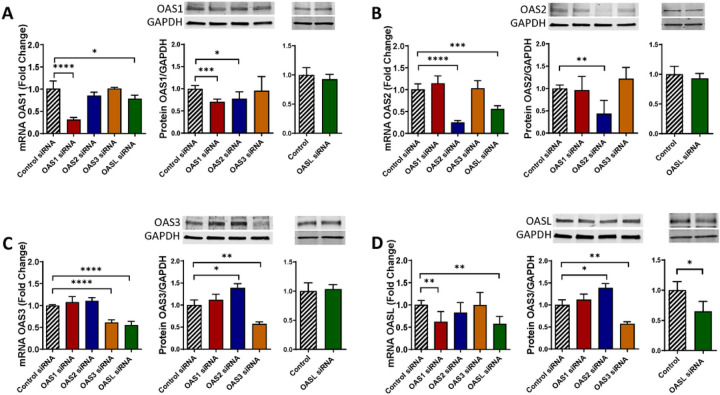
The OAS family members regulate each other’s expression. Pericytes were transfected as in Figure 4, and mRNA and protein levels of OAS1 **(A)** OAS2 **(B)** OAS3 **(C)** and OASL **(D)** was evaluated by q-PCR and immunoblotting, respectively. GAPDH was used as a housekeeping gene and loading control. Values are mean ± SEM. ****p < 0.0001, ***p = 0.0002, **p = 0.003, *p < 0.05, n = 4–6 independent samples per group.

The OAS family members may have overlapping functions in regulation of innate immunity; therefore, we examined whether they could interact among themselves and regulate each other’s expression. Such study has never been performed in the literature. Pericytes were transfected with control siRNA, OAS1 siRNA, OAS2 siRNA, OAS3 siRNA, or OASL siRNA, and mRNA and protein expression levels were measured for individual members of the OAS family. OAS1, OAS2 and OAS3 silencing significantly reduced OASL mRNA levels but not by other members of the OAS family (Fig. 6A–C) Furthermore, OASL silencing significantly decreased OAS1 mRNA levels; the effect, which did not apply to OAS2, or OAS3 (Fig. 6D).

At the protein level, downregulation of OAS1 decreased OAS2 expression, but no changes were found in OAS3 or OASL expression (Fig. 6A). OAS3 silencing increased the expression of OAS2 protein but it did not alter OAS1 or OASL protein levels (Fig. 6C). Furthermore, no changes were detected in the expression of any OAS members after OAS2 or OASL downregulation (Fig. 6B, D) These results indicate that individual members of the OAS family can influence each other expression; however, this input appears to be highly specific.

### HIV-1 infection alters the expression levels of the OAS genes and proteins

**Figure 7:**
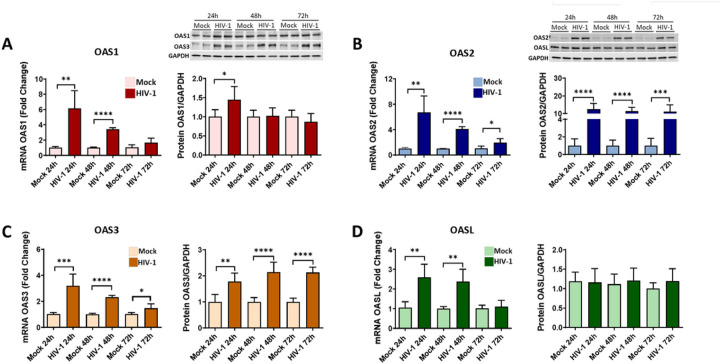
Impact of HIV-1 infection on OAS expression. Pericytes were either mock-infected or infected with 60 ng/mL HIV-1 p24 for 24, 48 or 72 hours and mRNA and protein expression of OAS1 **(A)**, OAS2 **(B)**, OAS3 **(C)** and OASL **(D)** was measured by q-PCR and immunoblotting, respectively. GAPDH was used as a housekeeping gene and loading control. Values are mean ± SEM. ****p < 0.0001, ***p = 0.0002, **p = 0.003, *p < 0.05, n = 4–6 independent samples per group.

The OAS family has been studied in viral infections and autoimmune disorders; however, only limited information is available on the role of these protein in HIV-infection [23, 50]. Moreover, no studies have defined the impact of the OAS gene family on HIV-1 infection in human brain pericytes. To investigate this relationship, pericytes were mock-infected or infected with HIV-1 for 24, 48 or 72 hours, and the mRNA and protein levels of individual members of the OAS family were evaluated. Infection with 60 ng/ml of HIV-1 for 24 or 48 hours resulted in a significantly increase in the expression of OAS1, OAS2, OAS3, and OASL at the mRNA level (Fig. 7A–D). Interestingly, this increase was gradually reduced over time, and only OAS2 and OAS3 showed a slight increase in mRNA expression after 72 hours of infection as compared to mock infection (Fig. 7B, C).

At the protein levels, there were notable differences in the response of individual OAS members to HIV-1 infection. The expression of OAS1 protein was increased only 24 hours after infection and returned to control levels after a longer infection period (Fig. 7A) There was a significant increase in the expression of OAS2 and OAS3 proteins 24, 48 or 72 hours post HIV-1 infection; however, these changes were more prominent for OAS 2 than those for OAS3 (Fig. 7B, C). Finally, no changes were detected in OASL protein levels after HIV-1 infection (Fig. 7D).

### Ocln regulates HIV-1 infection through an OAS-mediated mechanism

Studies from our laboratory have shown that human brain pericytes can regulate the extent of HIV-1 infection in various cell types, including brain pericytes [14, 16].

**Figure 8:**
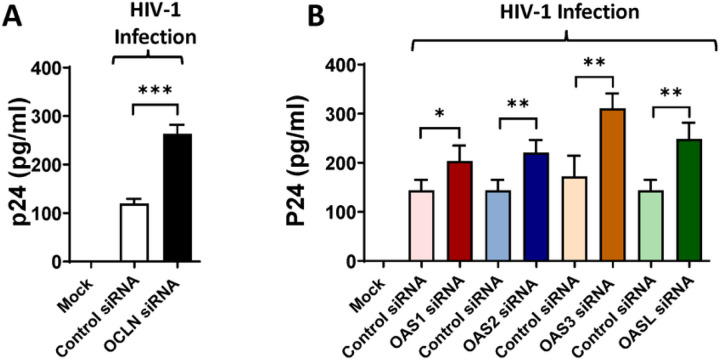
Ocln and OAS genes differentially regulate HIV-1 replication. Human brain pericytes were mock transfected or transfected with control siRNA **(A)** or with ocln siRNA, OAS1 siRNA, OAS2 siRNA, OAS3 siRNA or OASL siRNA **(B)**. Next, cells were either mock-infected or infected with 60 ng/ml HIV-1 p24 for 12 hours. p24 levels were analyzed 48h post infection in cell culture media by ELISA. Values are (Abderrazak, 2015 #94)mean ± SEM. ****p < 0.0001, ***p = 0.0002, **p = 0.003, *p < 0.0449, n = 4–10 per group.

Pericytes were transfected with ocln siRNA, OAS1 siRNA, OAS2 siRNA, OAS3 siRNA, or OASL siRNA and cultures were either mock-infected or infected with HIV-1 for 12 hours, followed by extensive washing to remove the unbound virus before addition of fresh medium. The levels of p24 antigen, the major structural component of HIV-1, were analyzed 48h after infection in the supernatants of cell cultures as the indicator of active HIV-1 replication. Downregulation of ocln resulted in increased p24 levels (Fig. 8A) Most interestingly, silencing of OAS1, OAS2, OAS3, or OASL markedly increased HIV-1 replication in human brain pericytes (Fig. 8B) providing the first evidence that the OAS family can regulate HIV-1 infection in human pericytes.

### HIV-1 infection of human brain pericytes does not affect RNaseL expression

**Figure 9:**
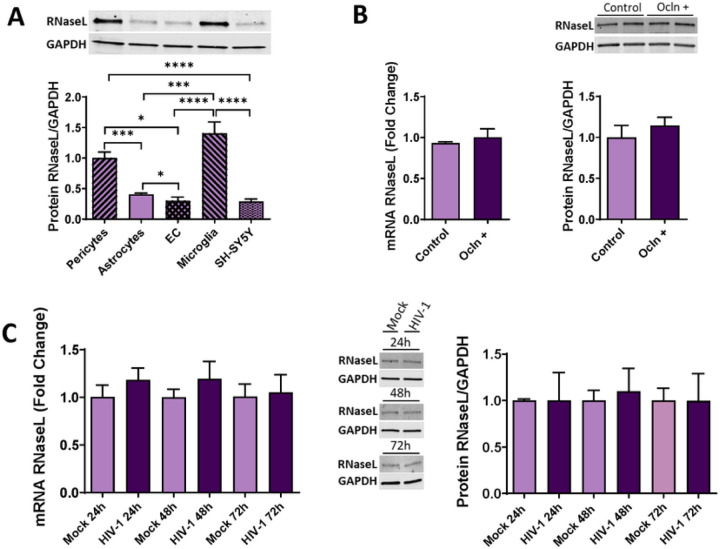
RNaseL expression pattern in cells of NVU. Expression of RNaseL in primary human brain pericytes, astrocytes, EC, immortalized human microglial cells, and SH-SY5Y neuroblastoma cell line as measured by immunoblotting **(A)** Pericytes were either mock-infected or infected as in Figure 6, and mRNA and protein expression of RNaseL was measured by qPCR and immunoblotting **(B)** Pericytes were transfected with ocln overexpressing vector PCMV3-OCLN or with PCMV3 control vector, and the expression of RNaseL was evaluated by qPCR and immunoblotting **(C)**. GAPDH was used as a housekeeping gene and loading control. Values are mean ± SEM. ****p < 0.0001, ***p = 0.0002, **p = 0.003, *p < 0.05, n = 4–6 independent samples per group.

Activated OAS can catalyze the oligomerization of ATP into 2’, 5- linked oligoadenylates (2–5A), which then activates RNaseL, one of the key elements of the OAS/RNaseL pathway by catalyzing ssRNA or rRNA [51]. Given the lack of information about RNaseL in human brain pericytes, we first aimed to characterize the expression of this endoribonuclease in individual cell types forming the NVU. Interestingly, pericytes along with microglia cells, express RnaseL to the highest extension, with a significantly lower expression in astrocytes, SH-SY5Y, and EC (Fig. 9A).

We next investigated whether RNaseL levels could be influenced by HIV-1 infection or by changes in ocln expression levels. No changes were found in the expression of RNaseL at mRNA or protein levels (Fig. 9C) after HIV-1 infection. Moreover, no changes were detected in RNaseL mRNA or protein levels after ocln overexpression (Fig. 9B).

## Discussion

Novel results of the present study provide evidence that the protein ocln can function as a regulator of host antiviral responses by controlling the OAS gene expression. The OAS/RNaseL system is considered to be one of the most important innate immunity pathways. The system acts via degradation of viral RNAs and was shown to be involved in protection against multiple viral infections [23, 27, 28, 52]. While the importance of this pathways has been demonstrated in immune responses in various cells types [23, 24], no studies had been perform on human brain pericytes and little is known about the expression and the role of OAS genes and proteins in human NVU cells.

Here we show for the first time that the members of the OAS family are expressed in all cells of the NVU; however, their expression differs between the studied cell types, indicating specific roles in protection against viral infections of the CNS (Fig. 1). OAS1, which is considered a “classical” ISG gene, exhibited the highest expression rates in pericytes and microglial cells, confirming the importance of these two cell types in innate immunity. While the role of microglia in neuroinflammation and/or protection against tissue injury is well recognized [53–55] the involvement of pericytes in innate immunity responses has been much less explored. However, our findings on a prominent expression of OAS1 in brain pericytes are consistent with the observation that these cells can harbor an active and potentially also latent HIV infection [6, 13, 16, 18].

The lowest expression of OAS1 was observed in astrocytes, a finding that was consistent with the prominent role of astrocytes in neuroinflammatory responses [56, 57]. Our studies also revealed a prominent expression of OAS2, OAS3, and OASL in SH-SY5Y cells; however, this is an immortalized neuroblastoma cell line, which does not fully represent mature neurons. Overall, differential expression pattern of the OAS proteins in individual cell types of the NVU offers new perspective on the role of the NVU in innate immunity protection at the level of the BBB.

Novel results of the present study demonstrate the role of ocln in controlling the expression of the members of the OAS family. Our interest in this topic was related to the discovery of metabolic and antiviral properties of ocln [14, 16, 22]. While it has been observed that IL-22 could simultaneously increase OAS1, OAS2 and ocln expression in end1/E6E7 cells [58], no literature reports evaluated a direct relationship between ocln and OAS expression. We observed that cellular ocln levels influenced the expression of all members of the OAS family at mRNA and protein levels. These effects were apparent as the results of both ocln overexpression and silencing (Figs. 3, 4). They also have strong clinical relevance as downregulation and/or upregulation of ocln levels is frequently found in neurological disorders such as stroke, neuroinflammation, or Alzheimer’s disease [59–61]. Importantly, they were specific for ocln as silencing of ZO-1, another TJ protein, did not affect the expression of the OAS genes. Interestingly, OASL expression exhibited several times higher upregulation after ocln overexpression compared to other OAS genes (Fig. 3). This interrelationship was mutual as OASL was the only member of the OAS family, which downregulation affected ocln expression (Fig. 5). We also identified that silencing OAS1, OAS2 or OAS3 resulted in a significant decrease in OASL mRNA levels; however, no other cross-interactions detected between the remaining OAS members (Fig. 6). These results are in line with the literature reports that OASL expression is differently regulated than the other OAS genes [62, 63].

The role of OASL gained significant attention in studies on innate and adaptive immune responses and it is considered to have a dual function in viral infection [63]. Human OASL contains two ubiquitin-like repeats and a CCY motif, whereas OAS1, OAS2, and OAS3, contain a CFK motif required for oligomerization. These structural differences are considered to be the cause of the lack of OAS activity of human OASL [30]. Interestingly, human OASL can bind to other proteins, such as RIG-1, and enhance RIG-1 antiviral responses [38]. Several studies have also hypothesized a competition of OASL with other OAS proteins by interfering with the 2–5A/RNaseL pathway, suggesting that OASL may negatively regulate OAS antiviral function. On the other hand, it was also shown that OASL could exhibit antiviral function against ssRNA viruses [64–66].

The expression of the OAS gene family is strongly associated not only with innate immunity and chronic infections but also with autoimmune diseases such as multiple sclerosis, rheumatoid arthritis, lupus erythematous and even with cancer [44–47, 67–70]. Recently, several OAS variants had been associated with COVID-19 severity [71–74], which is also in line with the data that pericytes are one of the target cells in SARS-CoV-2 infection [17]. Therefore, our results indicating that ocln can influence expression of the OAS genes may offer important implications for a better understanding of the pathology of autoimmune diseases and viral infections.

OAS belong to the ISG family; therefore, we investigated if IFNs are also expressed by pericytes and if their expression is influenced by ocln levels. Indeed, ocln modulation significantly impacted both the IFNa5 and the IFNp genes. In contrast, ocln did not affect the IFNa2 gene expression, indicating the specificity of responses (Fig. 2). Type I IFNs act via the IFNa/β membrane-associated receptors (IFNAR) 1 and 2 initiating a cross phosphorylation of JAK1 and TYK2 kinases [75, 76]. Activation of JAKs leads to a tyrosine phosphorylation of STAT1 and 2 [77]. As a result, STAT1 and STAT2 form a dimer that recruits IRF9 forming the heterotrimeric interferon-stimulated gene factor 3 (ISGF3) [78]. This complex translocates to the nucleus where it induces the transcription of several IRF and ISG genes [79–81]. Our results indicate for the first time that cellular ocln levels can modulate STAT1 expression at both the mRNA and protein levels. Moreover, to analyze the functional consequences of ocln upregulation we provide evidence that ocln overexpression leads to an increase in STAT1 phosphorylation at Tyr 701 and a higher levels of nucleus translocation (Fig. 2).

One of the main consequences of brain infection by HIV-1 is modification of the BBB integrity due to alterations in tight junction protein expression and the development of local inflammatory responses that may facilitate the transfer of the virus from the blood stream into the brain parenchyma [82, 83]. While the majority of HIV-1 replication in the brain appears to occur in microglial cells and perivascular macrophages [84, 85], other selected cell types of the BBB, such as astrocytes and brain pericytes can also be infected by HIV-1 [6, 12–17, 86]. A typical HIV infection in pericytes reveals a peak of viral replication in the initial phases of infection, followed by a gradual decline as measured by p24 levels. Importantly, these changes are associated with an increase in integrated HIV genome. Canonical HIV-1 infection uses both the main HIV-1 receptor, CD4, and the co-receptors, primarily CXCR4 and CCR5. Therefore, it is important that pericytes express high levels of HIV-1 co-receptors, CCR5 and CXCR4 as well as CD4, albeit at a lower level when compared to monocytic U937 cells [6, 13]. High expression of CCR5 and CXCR4 in brain pericytes makes them susceptible to both X4 and R5 tropic HIV-1 strains [13].

The OAS genes are induced by INFs and viral dsRNA. As the result of viral infection, there is an increase in OAS expression and activation which leads to synthesis of 2–5A from ATP and activation of RNaseL [23, 24, 87–89]. However, individual OAS proteins are characterized by different enzymatic parameters, subcellular location, and may have different roles [27–30]. Several recent studies had focused on OAS expression and polymorphism variants after virus infection. OAS1 is induced earlier than OAS2 and OAS3 during dengue virus infection [90], and mutations of the different OAS members had been correlated with several viral infections [91–94]. However, the exact impact of HIV-1 on OAS expression and the role of the OAS genes in HIV infection are well characterized and no studies had investigated the role of the OAS/RNaseL system in HIV infection of human brain pericytes. Our results reveal an increase in mRNA levels of all OAS genes 24 h post infection, i.e., at a time point, which corresponds with a peak of active HIV infection in brain pericytes. This initial increase was followed by a decline at 48 and 72 h post infection. In contrast to changes in mRNA levels, protein levels of OAS2 and OAS3 remained elevated in infected pericytes even after 48 and 72 h post infection, and OASL protein expression did not change as the result of infection (Fig. 7). These results suggest that upregulation of OAS2 and OAS3 is more sensitive to a lower HIV-1 viral load as compared to OAS1 in human brain pericytes. Indeed, the amount of viral RNA required for OAS3 activation was demonstrated to be lower than for the activation OAS 1 and OAS2 [95, 96].

Our study indicates that downregulation of the OAS genes can increase HIV-1 replication in human brain pericytes. These results are consistent with the reports on association between OAS activation and HIV regulation [97]. For example, induction of the OAS genes has been reported in the CNS of HIV-1 individuals [50]. Studies had also shown an increase in OAS/RNaseL expression in human macrophages after INF-Tau treatment [98]. An induction of RNaseL during HIV-1 infection and downregulation of the 2–5A/RNaseL pathway has been described in human T cells [99]. It was also observed that HIV-1 TAR RNA has an intrinsic ability to activate interferon-inducible enzymes [100] and that HIV-1 leader RNA activates dsRNA-dependent protein kinase and 2–5A-synthetase [101]. In contrast, it has been also reported that binding of Tat protein to HIV-1 TAR inhibits OAS activation [102]. Recently, more studies have been showing a relationship between the OAS/RNaseL pathway and HIV infection [50, 98]. Our results are also in line with the findings that treatment of HIV-infected cells with nuclease-resistant 2–5A^N6B^ lead to an HIV-1 inhibited replication [103].

Alterations of ocln expression have been linked to the regulatory mechanisms of HIV-1 infection *in vitro*. Specifically, a decrease in cellular ocln levels were shown to correlate with enhanced HIV-1 replication, and an opposing impact was observed upon overexpression of ocln [14, 16]. At least part of this effect was linked to ocln being a novel NADH oxidase that can control the expression and activation of the class-III histone deacetylase SIRT-1 and nuclear factor-κB [14]. Moreover, ocln can regulate HIV-1 budding from the infected cells by forming a complex with caveolin-1 and ALIX [16]. While the majority of research on the regulatory role of ocln on HIV infection was performed on brain pericytes [13, 14, 16], the findings were also confirmed in human primary macrophages, differentiated monocytic U937 cells, and HEK-293 cells [22]. Novel results in the current manuscript confirmed these findings and show that silencing of OAS1, OAS2, OAS3, or OASL markedly increased HIV-1 replication in human brain pericytes (Fig. 8).

One of the prominent antiviral pathways linked to the OAS family is RNaseL, which can induce antiviral protection through a combination of direct and indirect mechanisms that include viral ssRNA genome degradation, viral mRNA degradation of DNA and RNA viruses, as well as cellular mRNA and rRNA degradation. As the results of these events, activation of RNaseL can lead to apoptosis, reduced viral propagation, and enhancement of IFN production by activation of the RIG-1 antiviral responses, creating a positive feedback stimulation [38, 104–106]. RNaseL is also involved in RNA metabolism, autophagy, cell proliferation, cell differentiation and cancer [87, 107–110]. However, antiviral effects of RNaseL depend on the type of the virus and the cell types [27, 111–113]. Our results indicate that pericytes and microglia express the highest levels of RNaseL compared to other cell types of the NVU (Fig. 9). Nevertheless, infection with HIV-1 did not affect RNaseL expression in these cells (Fig. 9). In addition, modulation of pericyte ocln levels either by overexpression or silencing did not alter RNaseL expression (Fig. 9). The absence of RNaseL activation in HIV-1 infected pericytes is in line of studies that indicated that not all OAS antiviral functions are mediated by the RNaseL activation pathway [27, 28, 35, 114–116]. Thus, HIV-1 infection of human pericytes does not appear to involve RNaseL as an antiviral mechanism.

## Conclusions

**Figure 10:**
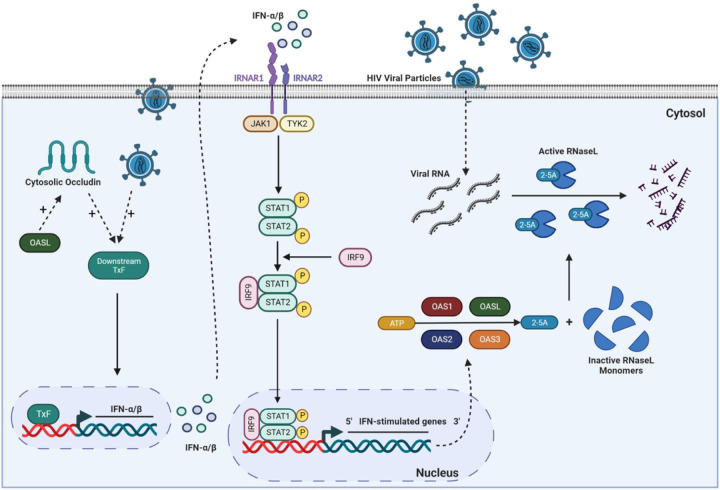
Proposed model of ocln-mediated regulation of the OAS genes and protection against HIV-1 infection in human brain pericytes. Ocln increases OAS1, OAS2, OAS3, and OASL expression levels by regulating the STAT signaling pathway. Our data indicate that ocln enhances STAT1 expression and phosphorylation levels. STAT1 and STAT2 form a dimer that recruits IRF9, also upregulated by ocln, and the complex moves to the nucleus where they bind to specific DNA elements, and initiate transcription of interferon stimulated OAS genes which restrain HIV replication. Furthermore, OASL can alter ocln expression in positive feedback. Following HIV-1 infection, there is also an increase in the expression of mRNA OAS1, OAS2, OAS3 and OASL and protein levels of OAS1, OAS2 and OAS3.TxF; transcription factor, IRNAR1; IFN receptor subunit 1, IRNAR2; IFN receptor subunit 2.

The present study described for the first time the expression pattern of the OAS family members and RNaseL in cells of the NVU. Mechanistically, we provided evidence that ocln can effectively modulate the OAS gene and protein expression. Importantly, we found that HIV-1 infection can differentially alter OAS expression levels and, in turn, modulation of the OAS genes can influence the rate of HIV-1 infection in human brain pericytes. Overall, our findings suggest that ocln is a critical regulator of immune response and viral infection regulation due to its ability to control INF-stimulated OAS expression levels (Fig. 10).

## Data Availability

All source data supporting the findings of this manuscript are available from the corresponding authors upon request.
